# Biochemical Properties of a Cold-Active Chitinase from Marine *Trichoderma gamsii* R1 and Its Application to Preparation of Chitin Oligosaccharides

**DOI:** 10.3390/md21060332

**Published:** 2023-05-29

**Authors:** Jianrong Wang, Mujin Zhu, Ping Wang, Wei Chen

**Affiliations:** 1 Shenzhen Raink Ecology & Environment Co., Ltd., Shenzhen 518102, China; 1934@noposion.com (M.Z.); 23525@noposion.com (P.W.); 24442@noposion.com (W.C.); 2 School of Food Science and Engineering, South China University of Technology, Guangzhou 510640, China

**Keywords:** *Trichoderma gamsii*, chitinase, cold-adapted, chitin oligosaccharides

## Abstract

The enzymatic degradation of different chitin polymers into chitin oligosaccharides (COSs) is of great significance given their better solubility and various biological applications. Chitinase plays a pivotal role in the enzymatic preparation of COSs. Herein, a cold-adapted and efficient chitinase (ChiTg) from the marine *Trichoderma gamsii* R1 was purified and characterized. The optimal temperature of ChiTg was 40 °C, and the relative activity at 5 °C was above 40.1%. Meanwhile, ChiTg was active and stable from pH 4.0 to 7.0. As an endo-type chitinase, ChiTg exhibited the highest activity with colloidal chitin, then with ball-milled and powdery chitin. In addition, ChiTg showed high efficiency when hydrolyzing colloidal chitin at different temperatures, and the end products were mainly composed of COSs with one to three degrees of polymerization. Furthermore, the results of bioinformatics analysis revealed that ChiTg belongs to the GH18 family, and its acidic surface and the flexible structure of its catalytic site may contribute to its high activity in cold conditions. The results of this study provide a cold-active and efficient chitinase and ideas for its application regarding the preparation of COSs from colloidal chitin.

## 1. Introduction

It is estimated that about 9.3 billion tons of crustaceans, such as crab, shrimp, and lobster, are produced annually, of which approximately 60% are inedible parts discarded as solid waste [1]. Previous studies have demonstrated that crustacean waste is mainly composed of chitin, protein, and minerals [2,3]. As an important constituent of crustacean waste, chitin is a linear polysaccharide that is composed of N-acetyl-D-glucosamine (GlcNAc) units linked by β-1,4 glycoside bonds. Meanwhile, chitin is also an important component of the cell walls of fungi and the exoskeletons of insects. Although chitin appears in abundance in the natural world, its poor solubility limits its further application [2,3]. Research studies have indicated that the derivatives of chitin, especially chitin oligosaccharides (COSs), exhibit better solubility and various kinds of biological activities such as antioxidant, immune-enhancing, and antimicrobial activities, which show great potential for application in different industries [4,5,6]. Chemical methods are usually chosen for the degradation of chitin or crustacean waste due to their simplicity and economic viability [7]. However, the disadvantages of these chemical methods, such as environmental pollution, harsh reaction conditions, and lack of control over the molecular structure of COSs, limit their further application [8]. Thus, it is essential to develop a specific, sustainable, and ecofriendly way to prepare COSs from chitin.

Recently, several studies have indicated that the enzymatic degradation of chitin displays the potential to replace the chemical methods owing to its mild reaction conditions, environmental friendliness, and providing the ability to control the process and final product [9,10]. As an important biocatalyst, chitinase can hydrolyze chitin by cleaving β-1,4 glycoside bonds and converting it into COSs with different degrees of polymerization. Previous studies have shown that chitinases have great potential value in various industries, with applications such as the preparation of COSs as biocontrol agents against pathogenic fungi and chitinous waste management [11,12]. Many chitinases have been isolated, purified, and characterized according to the origins from various organisms, including bacteria, fungi, and plants [11,12]. Generally, the chitinases from microorganisms are more suitable for industry applications than those from plants due to their excellent biochemical properties, extracellular secretion, and ease of cultivation [13]. In summary, the chitinases from microorganisms are mainly classified into two glycoside hydrolase (GH) families using the CAZy database: GH18 and GH19. Meanwhile, the chitinases from microorganisms can also be grouped as cold-adapted, mesophilic, and thermophilic based on their optimum reaction temperatures. Generally, cold-adapted enzymes offer some advantages for applications in different biotechnological processes. On one hand, cold-adapted enzymes exhibit high activity at low temperatures, so they can effectively reduce the energetic costs and risk of bacterial contamination. On the other hand, most cold-adapted enzymes are temperature-sensitive and easily inactivated by moderate heat. Compared with mesophilic or thermophilic chitinases, cold-active chitinases can remain active at low temperatures (0 to 10 °C) and exhibit several advantages such as the preparation of COSs at low temperatures, decreasing energy consumption, and biocontrol of microbial spoilage in refrigerated food, situations in which mesophilic or thermophilic chitinases often fail [14,15,16]. To date, several cold-active chitinases have been isolated and characterized, and most of them have usually been isolated from bacteria. It has been reported that chitinases EaChi39, Chi21702, AChi48, and ChiA from marine bacteria *Exiguobacterium antarcticum* DW2 [15], *Sanguibacter antarcticus* [17], *Pseudoalteromonas* sp. [18], and *Annona cherimola* [19] show high activity and efficiently catalyze at low temperatures. However, until now, few cold-adapted chitinases have been purified and characterized from fungi. 

*Trichoderma* spp. are well-known fungi that have been used as effective biocontrol agents against plant pathogens in agriculture due to their ability to secrete different types of extracellular enzymes, especially chitinases [20]. To date, many *Trichoderma* species have been identified as efficient producers for chitinases [20,21]. According to the literature, *Trichoderma* chitinases exhibit high efficiency toward pretreated chitin, suggesting their suitability for applications in the preparation of COSs [20,22]. Nevertheless, there are few reports regarding the purification and characterization of cold-adapted chitinase from *Trichoderma* species. 

In this work, a cold-adapted chitinase (ChiTg) from *T. gamsii* R1 was purified and characterized. Meanwhile, the gene encoding ChiTg was cloned and analyzed. To the best of our knowledge, this is the first cold-adapted chitinase to be characterized from *T. gamsii*, and it has great application value for the preparation of COSs. 

## 2. Results and Discussion

### 2.1. Purification and Identification of Chitinase from T. gamsii R1

**Figure 1:**
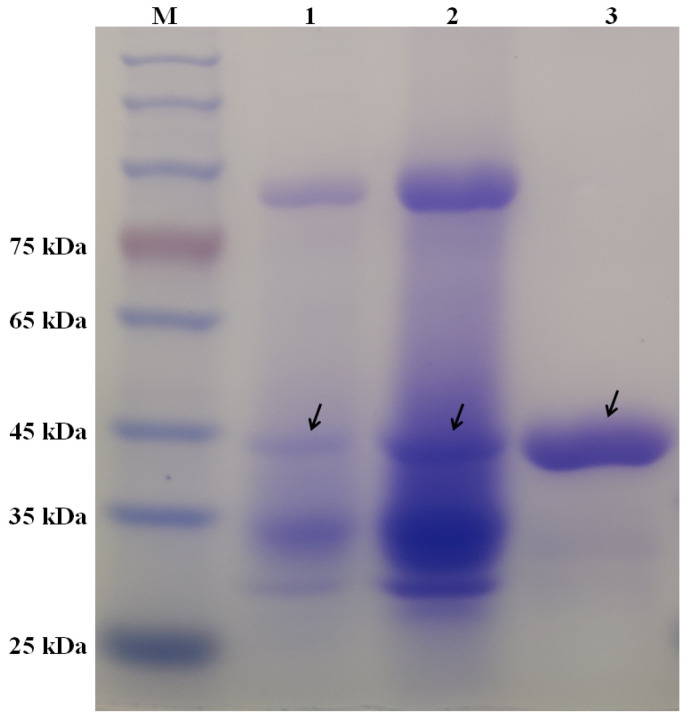
SDS-PAGE analysis of purified ChiTg. M: protein marker, 1: crude culture, 2: sample from ultrafiltration, and 3: purified ChiTg.

The fermentation conditions of *T. gamsii* R1 cultivated in shake flasks were optimized based on a single-factor experiment, and the results are shown in Supplementary Materials Figure S1. The culture supernatant with chitinase activity was centrifuged and further purified. As shown in Figure 1, a protein estimated at approximately 42 kDa was purified to apparent homogeneity after ultrafiltration and QSFF chromatography. In addition, the specific chitinase activity of a sample was 36.6 U/mg (Table 1). To date, several chitinases have been isolated from *Trichoderma* species such as Chit46 from *T. harzianum* GIM 3.442 [20], Ta-CHI42 from *T. asperellum* SH1 [23], chit42 from *T. aureoviride* M, etc. [24]. Briefly, the molecular masses of *Trichoderma* chitinases are in the range of 33 to 70 kDa, especially centered around 42 kDa [25,26]. Moreover, the *Trichoderma* chitinases with a molecular weight of 42 kDa have been extensively characterized and exhibit high-efficiency against pretreated chitin.

The obtained purified protein was further investigated via mass spectrometry. Several sequences of peptides were obtained, which included P1 (NFQPQNLVASDITHVIYS FMNFQADGTVVSGDAYADYQK), P2 (NLGLGGSMFWEASADKTGSDSLIGTSH R), P3 (VMLSIGGWTWSTNFPSAASTDANR), P4 (AGATVQYDSVAQAYYS YDPSSK), P5 (HYDDDSWNDVGNNAYGCVK), P6 (ASGYANAVYFTNWGIYGR), P7 (NLGLGG S MFWEASADK), and P8 (IVLGMPIYGR). The results of NCBIblastp revealed that these eight peptides exhibited high identity with the putative chitinase from *T. gamsii*. Therefore, the obtained purified protein was a chitinase and named ChiTg.

### 2.2. Substrate Specificity and Kinetic Parameters of ChiTg

The substrate preferences of ChiTg were determined with various substrates. As shown in Table 2, the preferred substrate for ChiTg was colloidal chitin, followed by ball-milled and powdery chitin. Due to pretreatment with hydrochloric acid, colloidal chitin is less crystalline and more easily accessible to chitinase than ball-milled and powdery chitin. It is particularly noteworthy that the preferred substrate for most of the characterized chitinases is colloidal chitin [9,20,27,28,29]. Meanwhile, ChiTg showed little activity against colloidal chitosan, with 85 to 95% DDA. In addition, ChiTg exhibited no activity with xylan or microcrystalline cellulose. 

Based on the substrate specificity, colloidal chitin was chosen as the substrate for the analysis of the kinetic parameters of ChiTg. The values of *K*_m_ and *V*_max_ were 0.47 mg/mL and 41.2 μmoL/min/mg, respectively. The values of *K*_m_ from different *Trichoderma* chitinases widely vary. For instance, the *K*_m_ of chitinases Chit46, ECH42, Tv-ECH1, and Chit42 from *T. harzianum* GIM 3.442 [20], *T. atroviride* [30], and *T. harzianum* CECT2413 [22] are 0.65, 1.9, and 1.7 mg/mL, respectively.

### 2.3. Effects of Temperature and pH on Activity and Stability of ChiTg

**Figure 2:**
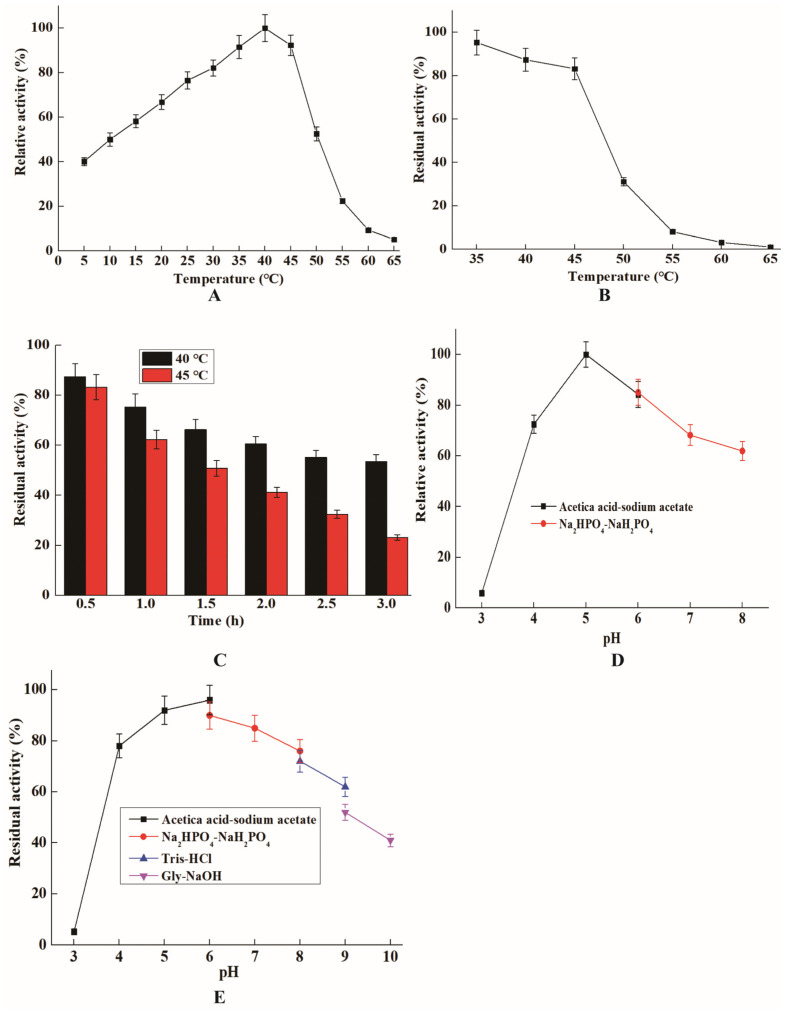
The characterization of purified ChiTg. Optimum temperature (**A**), thermostability (**B**), stability of ChiTg at 40 and 45 °C (**C**), optimum pH (**D**), and pH stability (**E**).

The purified ChiTg showed the maximum activity at 40 °C, which remained above 40.1% relative activity in the range of 5 to 45 °C (Figure 2A). Generally, the optimum temperatures of chitinases from different fungi are in the range of 30 to 70 °C. For instance, the chitinases from *T. harzianum* CECT2413, *M. thermophila* C1, and *H. grisea* ITCC 10,360.16 show maximum activity at 35, 55, and 70 °C, respectively [21,31,32]. As depicted in Figure 2A, the purified ChiTg was still active at 5 °C, and the relative activity was 40.2%, indicating ChiTg is a cold-active chitinase. It is particularly noteworthy that few chitinases from fungi have been reported to still exhibit high activity as the reaction temperature drops below 10 °C (Table 3). To date, cold-active chitinases have usually been isolated from marine bacteria, and few cold-active chitinases have been isolated from fungi [11]. Thus, it was meaningful to isolate and characterize ChiTg. Compared with its mesophilic and thermophilic counterparts, ChiTg is effective at quite low temperatures, so can reduce the energy cost for the preparation of COSs. 

As shown in Figure 2B, the residual activities of ChiTg incubation for 30 min at different temperatures (35 to 65 °C) were 95.3, 87.6, 83.2, 31.2, 8.1, 3.2, and 1.1%. In addition, the stability of ChiTg at 40 and 45 °C under different incubation times was further determined. The purified ChiTg was quite stable at 40 °C, and the residual activity was above 53.5%, even when the incubation time was 3 h (Figure 2C). Generally, cold-adapted chitinases display poor thermal stability due to their inherent structural flexibility, so they easily denature and lose activity as the temperature increases above 40 °C [38]. For instance, the chitinase ChiC from *Pseudoalteromonas* sp. DL-6 is only stable in the range of 10 to 30 °C, and the residual activity of chitinase ChiC rapidly decreases as the temperature increases above 40 °C [39]. Another chitinase ChiA, also isolated from *Pseudoalteromonas* sp. DL-6, retains above 50% of its maximum activity as the temperature falls below 40 °C [18]. Different from the above-mentioned chitinases, a cold-adapted chitinase EaChi39 from *Exiguobacterium antarcticum* DW2 exhibited excellent thermal stability even when the reaction temperature reached 60 °C [15]. The thermal stability results revealed that the reaction temperature for further application of ChiTg should be below 45 °C.

The effects of the pH on the activity and stability of ChiTg were also determined. ChiTg showed maximum activity at pH 5.0 and maintained a high relative activity in the range of pH 4.0 to 8.0 (Figure 2D). In addition, ChiTg was very stable when the reaction pH was in the range of 5.0 to 8.0, and the residual activity remained above 80%. When the reaction pH changed to 3.0 or above 9.0, the residual activity of ChiTg rapidly decreased (Figure 2E). The pH profile shows that ChiTg is suitable for the preparation of COSs from colloidal chitin because the process of the transformation of chitin to colloidal chitin occurs under acidic conditions.

### 2.4. Effects of Different Metal Ions on Stability of ChiTg

The relative activities of ChiTg treated with various metal ions were determined, and the results are shown in Supplementary Materials Table S1. ChiTg was slightly activated by Ca^2+^ and Zn^2+^, and the relative activities of ChiTg treated with those two metal ions were 104.3 and 106.5%, respectively. Meanwhile, the metal ions Mg^2+^, K^+^, Na^+^ and Cu^2+^ exhibited minimal inhibition on the activity of ChiTg, and the relative activities of ChiTg treated with those metal ions remained above 80.1%. Different from the above-mentioned metal ions, the activity of ChiTg was inhibited by Al^3+^ and Mn^2+^, which showed relative activities of 46.3 and 58.4%, respectively. Generally, the mineral salts of crustacean shells are mainly composed of Ca^2+^, Zn^2+^, and Mg^2+^. In this study, ChiTg was active and stable in the presence of Mg^2+^, Ca^2+^, and Zn^2+^, which would be helpful for further application for the recovery COSs from crustacean shells.

### 2.5. Hydrolytic Pattern of ChiTg

**Figure 3:**
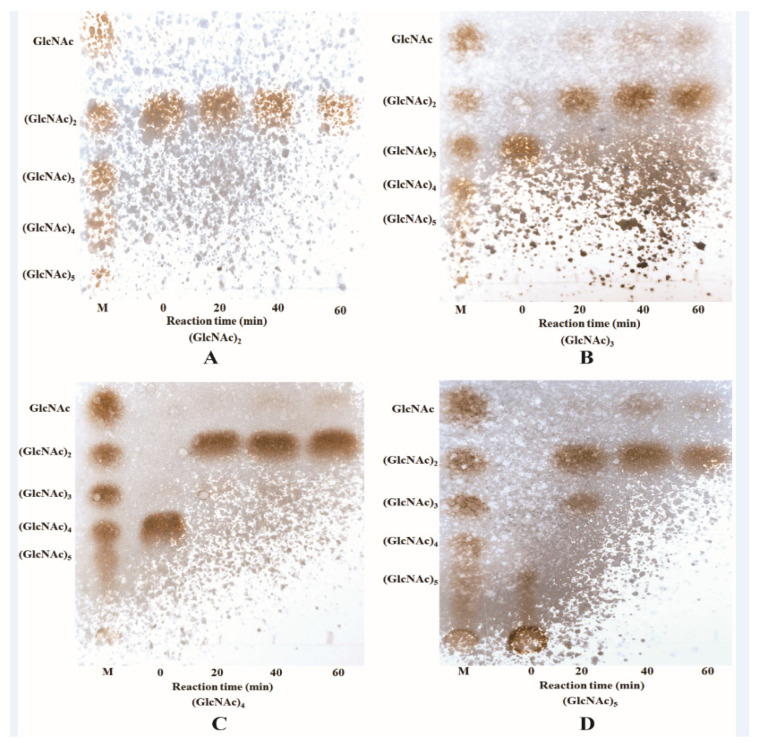
TLC analysis of the hydrolytic process of ChiTg toward (GlcNAc)_2_ (**A**), (GlcNAc)_3_ (**B**), (GlcNAc)_4_ (**C**), and (GlcNAc)_5_ (**D**).

The hydrolysis property of ChiTg was determined by using different COSs (DP 2 to 5) as substrates. ChiTg exhibited no activity toward (GlcNAc)_2,_ and (GlcNAc)_2_ was not transformed into smaller products by ChiTg even after 120 min of reaction (Figure 3A). Conversely, ChiTg hydrolyzed (GlcNAc)_3_ at a very fast rate, and most of the (GlcNAc)_3_ was cleaved and transformed to (GlcNAc)_2_ and GlcNAc as after 20 min of reaction (Figure 3B). In addition, ChiTg displayed high efficiency in hydrolyzing (GlcNAc)_4_ and (GlcNAc)_5_, which were almost fully decomposed and converted to smaller products when the reaction time reached 20 min. The reaction of ChiTg with (GlcNAc)_4_ generated (GlcNAc)_2_ as the end product (Figure 3C). (GlcNAc)_5_ was hydrolyzed and converted to (GlcNAc)_2_ and (GlcNAc)_3_ at a reaction time of 20 min (Figure 3D). When the reaction time reached 40 min, (GlcNAc)_3_ was decomposed and converted into (GlcNAc)_2_ and GlcNAc (Figure 3D). The end products of (GlcNAc)_5_ included (GlcNAc)_2_ and GlcNAc. 

The hydrolysis ability of ChiTg indicated that it had no hydrolytic activity against COSs with a DP below three, which is similar to that of previously reported chitinases from the GH18 or GH19 family, such as PxCHi52 from *Paenibacillus xylanexedens* Z2-4 [29], SaChiB from *Streptomyces alfalfa* [40], and CsCHiE from *Chitiniphilus shinanonensis* [41]. In addition, the different COSs hydrolyzed by ChiTg revealed that ChiTg is an endo-type chitinase. Furthermore, the hydrolytic pattern of ChiTg revealed its potential for application in the preparation of those COSs. 

### 2.6. Preparation of COSs from Colloidal Chitin

**Figure 4:**
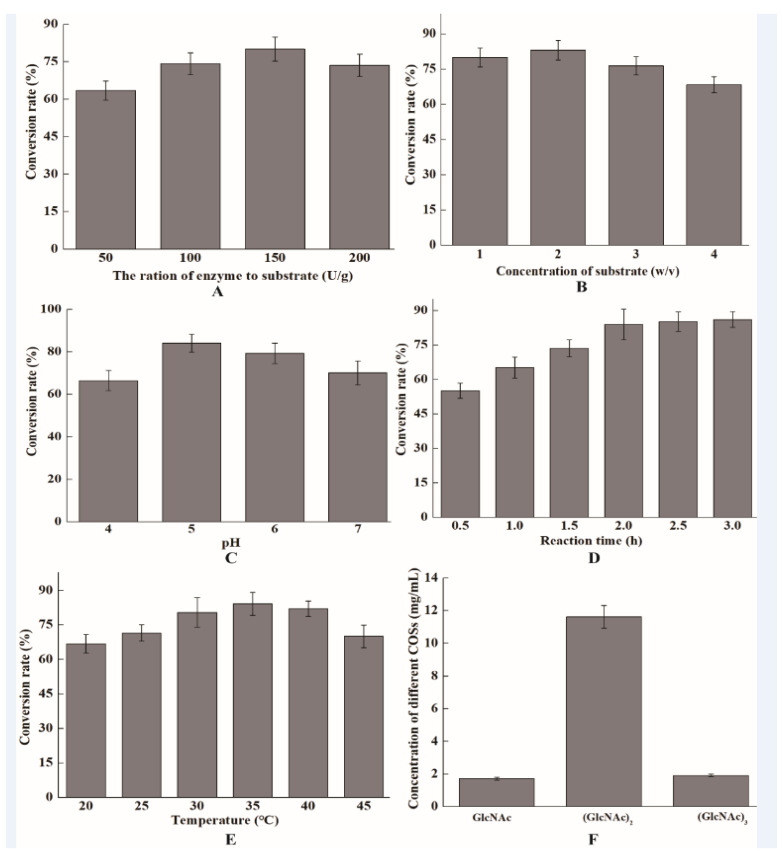
Preparation of COSs from colloidal chitin. Optimization of different ratios of enzyme to substrate (**A**), concentration of substrate (**B**), reaction pH (**C**), reaction time (**D**), and reaction temperature (**E**). HPLC analysis of hydrolysates from large-scale reaction (**F**).

Recently, COSs have attracted considerable attention due to their versatile functional properties, including their antioxidant, immunomodulatory, antimicrobial, and antitumor abilities [42,43]. Regarding substrate specificity, ChiTg displayed highest activity toward colloidal chitin. Therefore, colloidal chitin was used as the substrate for the preparation of COSs. One-single factor experiment was performed, and the results are depicted in Figure 4. The conversion rate gradually increased with the increasing addition of ChiTg, reaching the highest value as the ratio of enzyme to substrate was 150 U/g (Figure 4A). Additionally, the concentration of substrate was optimized, and the conversion rates of groups 1 to 4% (*w/v*) were 80.1, 83.2, 76.5, and 68.5%, respectively (Figure 4B). Furthermore, the effects of reaction pH, time, and temperature on the conversion rate were investigated. As shown in Figure 4C, ChiTg exhibited the best hydrolysis efficiency on colloidal chitin when the reaction pH was 5.0, followed by when the pH was 6.0 and 4.0, which is in accordance with the pH profile of ChiTg. As can be seen from Figure 4D, the conversion rate quickly increases from 0.5 to 2 h of reaction time. However, the conversion rate slowly increased when the reaction duration was above 2 h. As depicted in Figure 4E, the optimal temperature for the ChiTg degradation of colloidal chitin to COSs was 35 °C, followed by 40 and 30 °C. 

Based on the results of the above one-single factor experiment, a large-scale reaction was performed under the optimum reaction conditions. Most of the insoluble colloidal chitin was converted to soluble COSs after 2 h of reaction. The hydrolysates mainly included GlcNAc, (GlcNAc)_2_, and (GlcNAc)_3_; and the concentrations of those three COSs were 1.73, 11.62, and 1.92 mg/mL, respectively (Figure 4F). The HPLC charts of the hydrolysates are shown in Supplementary Materials Figure S2. In addition, the conversion rate was 84.2% after 2 h of reaction. Recently, several chitinases from different GH families have been used to prepare COSs from different chitin polymers. For instance, a chitinase, rChit46, from *T. harzianum* GIM 3.442 hydrolyzed 80.5% of 2% (*w/v*) colloidal chitin into GlcNAc, (GlcNAc)_2_, (GlcNAc)_3_, and (GlcNAc)_4_ after 3 h reaction at 45 °C in the presence of 200 U/mL of rChit46 [20]. In addition, Song et al. (2020) reported a chitinase, ChiA, from *Bacillus licheniformis* WX-02 can hydrolyze 10% (*w/v*) colloidal chitin into COSs at 50 °C and the conversion rate is 89% after 12 h reaction [44]. Zhang et al. (2021) found that a chitinase ChiA from *Paenibacillus xylanexedens* Z2-4 could convert about 60% of 1% (*w/v*) colloidal chitin into GlcNAc and (GlcNAc)_2_ after 24 h reaction at 45 °C [29]. In this study, ChiTg efficiently prepared COSs from colloidal chitin at lower temperatures (20 to 35 °C) with a higher conversion rate, indicating that ChiTg has great potential and is competitive for the preparation of COSs.

### 2.7. Gene Cloning and Bioinformatics Analysis of ChiTg

**Figure 5:**
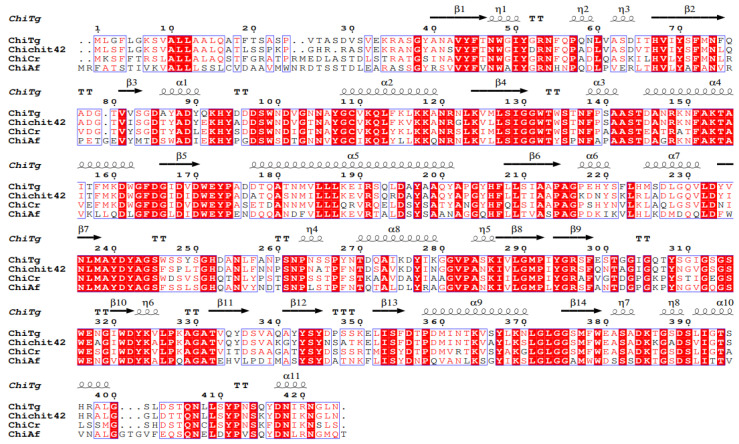
Sequence alignment of ChiTg with already crystallized chitinase. The listed sequences include Chit42 from *Trichoderma harzianum* (S78423.1), ChiCr from *Clonostachys rosea* (ABV57861.1), and ChiAf from *Aspergillus fumigatus* Af293 (XP_747065.1).

In order to better understand ChiTg, the gene encoding ChiTg was obtained via RT-PCR, and the bioinformatics analysis of ChiTg was performed. The results of the sequencing and prediction of the open reading frame indicated that *chitg* is 1275 bp in length, encoding 424 amino acids. Based on the results of NCBIblastn, we deduced that ChiTg belongs to the GH18 family due to its high sequence identity with other GH18 family chitinases from *T. harzianum* (accession No. AAA98644.1) and *T. atroviride* (accession No. BAB40593.1). In addition, the results of ProtParam analysis showed that 36 and 19 residues of ChiTg were negatively (Asp and Glu) and positively charged (Arg and Lys), respectively. Moreover, the first 27 amino acid residues of ChiTg were signal peptides. The results of the multiple alignment of ChiTg against already crystallized chitinase indicated that ChiTg also contains the classic motif (DXDXE) of the GH18 family. Meanwhile, three amino acids (D146, D148, and E150) were considered as the catalytic residues (Figure 5).

**Figure 6:**
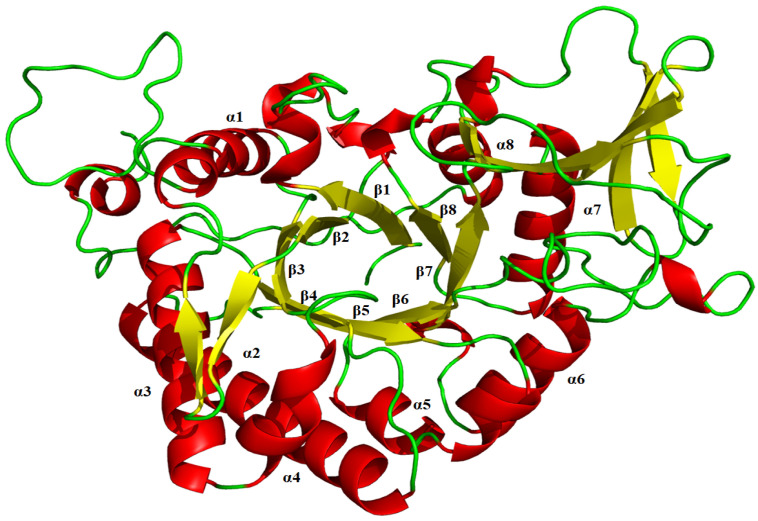
The overall structure of ChiTg.

**Figure 7:**
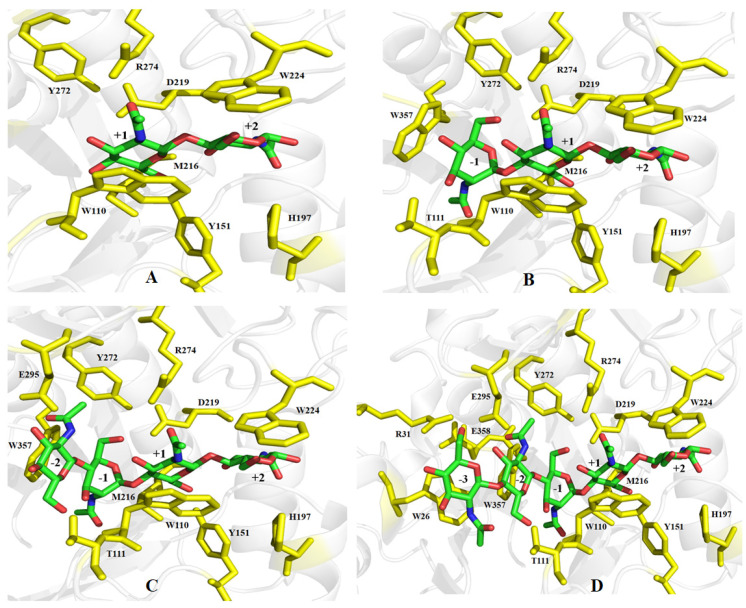
Homology modeling structure of ChiTg. Interactions between ChiTg and (GlcNAc)_2_ (**A**), (GlcNAc)_3_ (**B**), (GlcNAc)_4_ (**C**), and (GlcNAc)_5_ (**D**). The amino acids residues colored in yellow play an important role in catalytic hydrolysis of substrates.

The results of NCBIblastn revealed that the sequence identity of ChiTg with chitinase Chit42 from *T. harzianum* CECT2413 is 82.5%. Then, the three-dimensional structure of ChiTg was obtained by homology modeling using already crystallized chitinase Chit42 from *T. harzianum* CECT2413 as a template (PDB deposition: 6ylj.1). As shown in Figure 6, the overall structure of ChiTg is characterized as an (α/β) TIM-barrel topology, which is mainly composed of eight β strands (β1 to β8) and eight α helices (α1 to α8). Different from the already reported chitinases, ChiTg contains an additional chitinase insertion domain that plays crucial roles in substrate binding and catalysis. Meanwhile, the docking results revealed that hydrogen bonds and hydrophobic interaction were the main forces through which ChiTg recognizes, binds, and catalyzes different substrates. In addition, several amino acids (W26, R52, G30, R31, D63, W110, T111, D146, D148, E150, Y151, H197, Y218, D219, Y220, W224, Y246, Y272, R274, E295, and I298) may be related to the catalytic hydrolysis of the substrate (Figure 7A–D). Furthermore, the docking results displayed that (GlcNAc)_3_ is the smallest substrate for ChiTg, and (GlcNAc)_2_ is the main end product, which is in accordance with the results of the hydrolytic pattern of ChiTg (Figure 3).

**Figure 8:**
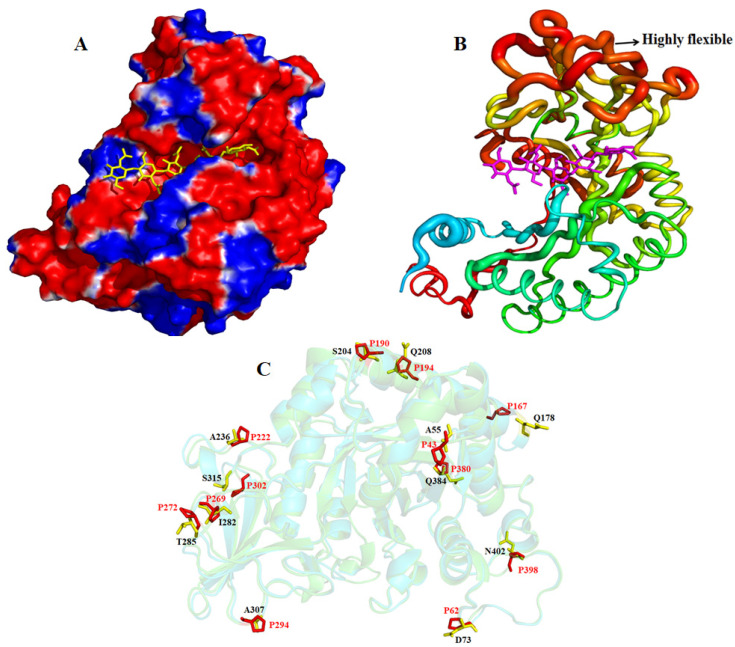
The potential factors related to the cold adaptation of ChiTg. Surface electrostatic potential of ChiTg (**A**). The surface coloring is based on the electrostatic potential, with a gradient from red (electronegative) to blue (electropositive). B-factor changes over the backbone of ChiTg (**B**). Tertiary structure alignment between ChiTg and thermostable chitinase Chit1 from *Thermomyces lanuginosu s*(**C**). The amino acid residues on the surface colored in red and yellow are from Chit1 and ChiTg, respectively.

It is particularly noteworthy that the ability of cold-adapted enzymes to remain active at cold conditions is related to their structure [11,14]. Generally, the molecular mechanisms of cold-adapted enzymes are achieved through their highly acidic surface, low levels of rigidifying residues, small number of hydrogen bonds, low salt bridge content, having fewer hydrophobic interactions, and the flexible structure of catalytic site [16,38,45,46,47,48,49]. The cold adaptation ability of ChiTg may relate to its acidic surface and the flexible structure of its catalytic site (Figure 8A,B). A total of 36 residues of ChiTg are negatively charged (Asp and Glu), most of which are located at the surface (Figure 8A). A previous study indicated that acidic surfaces are helpful for retaining the solvation of cold-adapted enzymes, as water tends to be more viscous at lower temperature [45]. Additionally, the B factors showed that the additional chitinase insertion domain of ChiTg is highly flexible (Figure 8B), which is beneficial for substrate binding at low temperatures. Generally, the catalytic region of cold-adaptive enzymes is more flexible than their mesophilic and thermophilic counterparts. Research has shown that the higher flexibility of the catalytic site of cold-adapted enzymes is helpful for the formation of enzyme–substrate complexes at lower energy at lower temperatures [45,50]. Moreover, the tertiary structure alignment between ChiTg and a thermostable chitinase, Chit1 [37], from *Thermomyces lanuginosus* revealed that amino acid substitution may also be related to cold tolerance (Figure 8C). Compared with ChiTg, the surface of thermostable chitinase Chit1 has more proline, which is helpful for increasing thermal stability (Figure 8C). 

## 3. Materials and Methods

### 3.1. Strains and Materials

*T. gamsii* R1 was isolated from shrimp shell waste and identified via ITS sequencing and then conserved in our laboratory. *E. coli* Dh5α competent cells were purchased from Tiangen Biotechnology (Beijing, China). PrimeSTAR^®^ HS (Premix) DNA polymerase and PrimeScript™ RT-PCR Kits were purchased from Takara Biotechnology (Beijing, China). COSs with different degrees of polymerization (DPs), including N-acetyl-D-glucosamine (DP1), N-acetyl-chitobiose (DP2), N-acetyl-chitotriose (DP3), N-acetyl-chitotetraose (DP4), and N-acetyl-chitopentaose (DP5), were purchased from Qingdao BZ Oligo Biotech (Qingdao, China). Furthermore, GlcNAc, (GlcNAc)_2_, (GlcNAc)_3_, (GlcNAc)_4_, and (GlcNAc)_5_ represent N-acetyl-D-glucosamine, N-acetyl-chitobiose, N-acetyl-chitotriose, N-acetyl-chitotetraose, and N-acetyl-chitopentaose, respectively. Powdery chitin, xylan, microcrystalline cellulose, and chitosan with a degree of deacetylation (DDA) of 85 to 95% were purchased from Yuanye Biotechnology (Shanghai, China). The colloidal and ball milled-chitin were prepared according to a previously described method [51]. 

### 3.2. Production of Chitinase from T. gamsii R1 in Shake Flasks

A single-factor design was used to optimize the production of chitinase from *T. gamsii* R1, which included different nitrogen sources (yeast extract, peptone, or ammonium sulfate), concentrations of colloidal chitin (0.5 to 2%, *w/v*), fermentation temperatures (25 to 35 °C), and times (3 to 5 d). After single-factor experiment optimization, *T. gamsii* R1 was inoculated into 100 mL of fermentation medium (1.5% peptone, 1.0% colloidal chitin, 0.1% MgSO_4_•7H_2_O, 0.05%, FeSO4•7H_2_O, and 0.3% K_2_HPO_4_, *w/v*) in 500 mL flasks and incubated at 200 rpm and 30 °C for 4 d. The fermentation culture was centrifuged at 10,000× *g* and 6 °C for 10 min, and the obtained supernatant was used for a chitinase activity assay and purification.

### 3.3. Enzyme Activity Assay

The chitinase activity was determined according to a method described in a previous study [9]. We used 1% (*w/v*) colloidal chitin (pH 5.0) as the substrate. In the first step, 200 µL of diluted enzyme was added to a 2 mL test tube containing 200 µL of colloidal chitin and incubated at 40 °C for 30 min. In the second step, the reaction was stopped by the addition of 600 µL of 3,5-dinitrosalicylic acid (DNS), which was then incubated at 100 °C for 10 min. Finally, the cool-down solution was centrifuged at 11,000× *g* for 5 min, and the supernatant was monitored at 540 nm with a spectrophotometer. The reactions with heat-denatured enzyme were used as the control. One unit of enzyme activity was defined as the amount of enzyme that releases 1 µmol of reducing sugars per minute.

### 3.4. Enzyme Purification and Peptide Sequence Analysis

The process for enzyme purification was similar to that described in a previous study [15]. To begin, the obtained supernatant was concentrated via ultrafiltration with a membrane with a 10 kDa cut-off (Merck Millipore, Billerica, MA, USA). Then, the obtained protein samples were purified via Q Sepharase Fast Flow chromatography (GE life science, Fairfield, CT, USA). Finally, the homogenous fractions were pooled and concentrated via ultrafiltration with a membrane with a 10 kDa cut-off. The purified protein samples were analyzed with a chitinase activity assay and SDS-PAGE. The purified protein sample with chitinase activity was named ChiTg. 

The partial amino acid sequence of ChiTg was analyzed via LC-MS/MS with a Q Exactive Orbitrap HF mass spectrometer with a nanoelectrospray ionization source (ThermoFisher Scientific, Waltham, MA, USA) and an LC-20AD nano high-performance liquid chromatography (HPLC) system equipped with a MonoCap C18 trap column (0.2 × 50 mm, 5 μm; Shimadzu, Kyoto, Japan). The detailed protocol is provided in the Supplementary Materials. 

### 3.5. Kinetic Parameters of Purified ChiTg

The method for the determination of the kinetic parameters was similar to a previously reported method [52]. Different concentrations of colloidal chitin (1 to 10 mg/mL) were used as the substrate. The values of *K*_m_ and *V*_max_ were calculated with GraphPad Prism 8.0.2.

### 3.6. Effects of pH and Temperature on Activity and Stability of ChiTg

The method we used for the determination of the effects of pH and temperature on the activity and stability of the purified ChiTg was same as that reported in a previous study [53]. For the optimum pH, the activity of purified ChiTg was detected from pH 4.0 to 7.0, and the pH with the highest activity was set to 100%. For pH stability, the purified ChiTg was diluted in different buffers with pH ranging from 4.0 to 10.0, and the sample without any treatment was set as the control. 

The optimum temperature of the purified ChiTg was determined within 30 to 70 °C, and the temperature with the highest activity was set to 100%. Regarding thermal stability, the purified ChiTg was incubated at temperatures ranging from 40 to 60 °C for 30 min, and the sample without any treatment was set as the control. In addition, the thermal stability of purified ChiTg at 40 and 45 °C treated for different durations was also determined.

### 3.7. Effects of Different Metal Ions on Stability of ChiTg

The effects of different metal ions (Mg^2+^, K^+^, Co^2+^, Cu^2+^, Zn^2+^, Ca^2+^, Na^+^, Mn^2+^, and Al^3+^) on the on the stability of purified ChiTg were determined by incubating enzyme samples for 4 h at 25 °C. The enzyme activity of purified ChiTg without metal ions was considered as 100%.

### 3.8. Hydrolysis Properties of ChiTg

The hydrolysis ability of recombinant ChiTg was determined based on the methods in a previous study using different COSs as the substrate [29]. The reaction was carried out as follows: to begin with, the purified recombinant ChiTg was mixed with different COSs (1%, *w/v*). Then, the mixture was incubated at 40 °C for different durations. Finally, the samples withdrawn at different times were incubated at 80 °C for 5 min and used for thin-layer chromatography (TLC) analysis. The process for TLC analysis was same as that previously reported [40]. 

### 3.9. Substrate Specificity of ChiTg

The method followed for the determination of substrate specificity of ChiTg was the same as that previously reported [41]. Colloidal chitosan with different DDAs (85 and 95%); colloidal, ball- milled, and powdery chitin; pretreated shrimp shell powder; xylan; and microcrystalline cellulose were used as the substrates for the activity assay. The substrate with highest activity was set to 100%.

### 3.10. Preparation of COSs from Colloidal Chitin

A single-factor design was used to optimize the preparation of COSs from colloidal chitin. Different ratios of enzyme to substrate (50, 100, 150, and 200 U/g), concentrations of substrate (1, 2, 3, and 4%, *w/v*), reaction pH values (4, 5, 6, and 7), reaction times (0.5, 1, 1.5, 2, 2.5, and 3 h), and reaction temperatures (20, 25, 30, 35, 40, and 45 °C) were selected for optimization. Reactions were carried out in a 50 mL flask containing 10 mL of colloidal chitin, which were incubated in a shaker at 150 rpm. The reaction was ended by incubating at 70 °C for 5 min and then centrifuging at 10,000× *g* and 10 °C for 10 min. The residual colloidal chitin was dried out at 90 °C. The conversion rate was calculated according to the method described in a previous study [20]. 

A large-scale reaction (1 L flask containing 200 mL of colloidal chitin) for the preparation of COSs was performed under the optimum reaction conditions. The ratio of enzyme to substrate; concentration of substrate; reaction pH, time, and temperature of the large-scale reaction were 150 U/g, 2%, 5.0, 2 h, and 35 °C, respectively. After 2 h of reaction, the end products were identified via high-performance liquid chromatography (HPLC). The HPLC system was the same as that in a previous study [54]. The mobile phase was composed of acetonitrile and water (70:30, *v/v*), and the flow rate was 0.8 mL/ min. Finally, the concentrations of different COSs were quantified by integrating the peak areas according to the respective standard curve.

### 3.11. Gene Cloning and Bioinformatics Analysis of ChiTg

*T. gamsii* R1 was cultivated on fermentation medium at 30 °C for 72 h. Then, the mycelia of *T. gamsii* R1 were collected and used for the extraction of total RNA. The total RNA was isolated with an E.Z.N.A.TM Fungal RNA Kit (Omega, Guangzhou, China). After that, the cDNA was synthesized with a PrimeScript 1st-strand cDNA synthesis kit (Takara, Beijing, China). Based on the hypothetical chitinase sequence from *T. gamsii* T6085 (GenBank: XM_018810913), two primers (Tg-fw: 5′-ATGTTGGGTTTCCTCGGA AAG-3′ and Tg-rev: 5′-TTAGTTAAGACCGTTTCGGAT-3′) were designed and used for PCR amplification. The obtained PCR product was purified, ligated to pMD20T, and sequenced.

NCBIblastn and NCBIblastp were used to analyze the sequence identity of ChiTg and ChiTg against different chitinases. The physical and chemical parameters of ChiTg were predicted with ProtParam (1.0). The prediction of the signal peptides of ChiTg was performed with the SignalP 5.0 server. The homology modeling of ChiTg was performed with SWISS-MODEL. The interaction between different COSs (DP 2 to 5) and ChiTg was performed with Autodock vina. Pymol1.8.x was used to analyze of the three-dimensional structure of ChiTg.

### 3.12. Statistical Analysis

All measurements were performed in triplicate. The data were analyzed with Statistical Package for the Social Sciences (SPSS) software (version 18.0, SPSS Inc., Amunk, NY, USA), and the results are expressed as mean ± standard deviation (SD).

## 4. Conclusions

In conclusion, a cold-active and efficient chitinase from *T. gamsii* R1 was successfully purified and characterized. In addition, the gene encoding ChiTg was cloned, and the bioinformatics analysis of ChiTg was performed. ChiTg belongs to the GH18 family and remains active and stable at temperatures of 5 to 40 °C and pH values of 4.0 to 7.0. As an endo-type chitinase, ChiTg displays activity toward different chitin polymers such as colloidal, ball mill, and powdery chitin. Moreover, ChiTg highly efficiently hydrolyzes colloidal chitin in the preparation of COSs. Overall, the excellent properties of ChiTg reveal its high value for the future bioconversion of different chitin polymers into COSs.

## Figures and Tables

**Table 1:** Purification of ChiTg from *T. gamsii* R1.

Purification Step	Total Activity (U)	Protein (mg)	Specific Activity (U/mg)	Purification Factor (fold)	Recovery (%)
Crude culture	1076.8 ± 42.5	468.2 ± 22.1	2.3 ± 0.1	1	100
Ultrafiltration	764.2 ± 35.2	201.1 ± 9.5	3.8 ± 0.2	1.65	71
QSFF chromatography	204.6 ± 12.3	5.6 ± 0.3	36.6 ± 1.6	16.8	19

**Table 2:** The substrate specificity of ChiTg.

Substrate	Activity (U/mg)
Powdery chitin	5.7 ± 0.2
Colloidal chitin	36.6 ± 1.8
ball milled chitin	27.9 ± 1.6
xylan	ND *
microcrystalline cellulose	ND
Colloidal chitosan with 85% DDA	1.1 ± 0.1
Colloidal chitosan with 90% DDA	1.3 ± 0.1
Colloidal chitosan with 95% DDA	1.3 ± 0.1
pretreated shrimp shell powder	4.5 ± 0.3

* ND, enzyme activity was not detected.

**Table 3:** Temperature properties of chitinases from different fungi.

Microorganism	Optimum Temperature (°C)	Relative Activity at 5 °C (%)	Relative Activity at 10 °C (%)	Relative Activity at 30 °C (%)	Reference
*T. gamsii* R1	40	40.2	50.1	82.1	This study
*T. harzianum* GIM 3.442	45	NR *	NR	about 48%	[20]
*T. harzianum* CECT2413	35	NR	NR	about 88%	[22]
*T. asperellum* SH16	45	NR	NR	about 62%	[23]
*T. asperellum* PQ34	45	NR	NR	about 60%	[33]
*A. niger* CBS 513.88	40	NR	NR	about 83%	[34]
*T. harzianum* ThHP3	60	about 20%	about 23%	about 62%	[31]
*Myceliophthora thermophila* C1	55	NR	NR	about 31%	[32]
*H. grisea* ITCC 10,360.16	70	NR	NR	NR	[35]
*Penicillium oxalicum* k10	40	NR	NR	about 62%	[36]
*Aspergillus fumigatus* df347	45	NR	NR	about 47%	[27]
*Thermomyces lanuginosus SSBP*	50	NR	NR	NR	[37]

* NR, relative activity at this temperature was not reported.

## Data Availability

Data are contained within the article or Supplementary Material.

## Supplementary Materials

The following supporting information can be downloaded at: https://www.mdpi.com/article/10.3390/md21060332/s1, Figure S1: Single-factor optimization for production of chitinase from *T. gamsii* R1; Figure S2: HPLC charts of hydrolysates; Table S1: Effects of different metal ions on stability of ChiTg.
