# Supplementary material for: Biochemical Properties of a Cold-Active Chitinase from Marine Trichoderma gamsii R1 and Its Application to Preparation of Chitin Oligosaccharides
Source: Mar Drugs. 2023 May 29;21(6):332. doi: 10.3390/md21060332 (PMC10302006; doi:10.3390/md21060332)
Supplement: Supplementary file 1 [file marinedrugs-21-00332-s001.zip › marinedrugs-2407170-supplementary.pdf]

1. LC-MS/MS analysis of partial amino acid sequence of ChiTg

The target protein bands in the stained SDS-PAGE gels were manually excised and de-stained by acetonitrile (50%), then treated with dithiothreitol (10 mM) and iodoa-cetamide (55 mM) and finally digested overnight at 37 °C with sequencing-grade trypsin. The digested solutions were subjected to peptide sequencing analysis by LC-MS/MS with a Q Exactive Orbitrap HF mass spectrometer with a nanoelectrospray ionisation source (ThermoFisher Scientific, USA) and an LC-20AD nano high-performance liquid chromatography (HPLC) system equipped with a MonoCap C18 trap column (0.2 × 50 mm, 5 μm; Shimadzu, Japan).

The HPLC mobile phase comprised solutions A (5% acetonitrile containing 0.1% formic acid) and B (95% acetonitrile containing 0.1% formic acid). Samples were loaded by mobile phase A at a flow rate of 8 μL min^{-1} for 4 min. The gradient elution program was as follows: 0 min, 2% B; 40 min, 35% B; 45 min, 80% B; 49 min, 80% B; 50 min, 2% B; and 60 min, 2% B at a flow rate of 300 nL•min^{-1}.

The resulting nanoflow liquid chromatography eluate was directly subjected to nanoelectrospray ionization, followed by the mass spectrometry in the positive-ion mode. The electrospray voltage and capillary temperature were 1.6 kV and 250 °C, respectively. The MS scan was acquired within an m/z range of 350 to 2,000 in the Orbitrap at a resolution of 70,000. The MS/MS scans were detected in a high-energy collisional dissociation operating mode with 27.0% normalised collision energy. Identical ions that were detected more than two times within 15 s were dynamically excluded.

The collected MS/MS profiles were analysed with Proteome Discoverer software (ThermoFisher Scientific, USA) and searched for in the National Center for Biotechnology Information (NCBI) protein database using the following search parameters: fixed modi-fications, carbamidomethyl (C); variable modifications, oxidation (M); enzyme, trypsin; maximum missed cleavages, 2; peptide mass tolerance, 20 ppm; fragment mass tolerance, 0.6 Da; mass values, monoisotopic.

1. Supplement Table

Table S1. Effects of different metal ions on the activity of ChiTg.

Substrate	Relative activity (%)
Ca^{2+}	104.3
Mg^{2+}	97.3
Zn^{2+}	106.5
K^{+}	89.6
Na^{+}	92.3
Cu^{2+}	81.2
Al^{3+}	46.3
Mn^{2+}	58.4

2. Supplement Figures

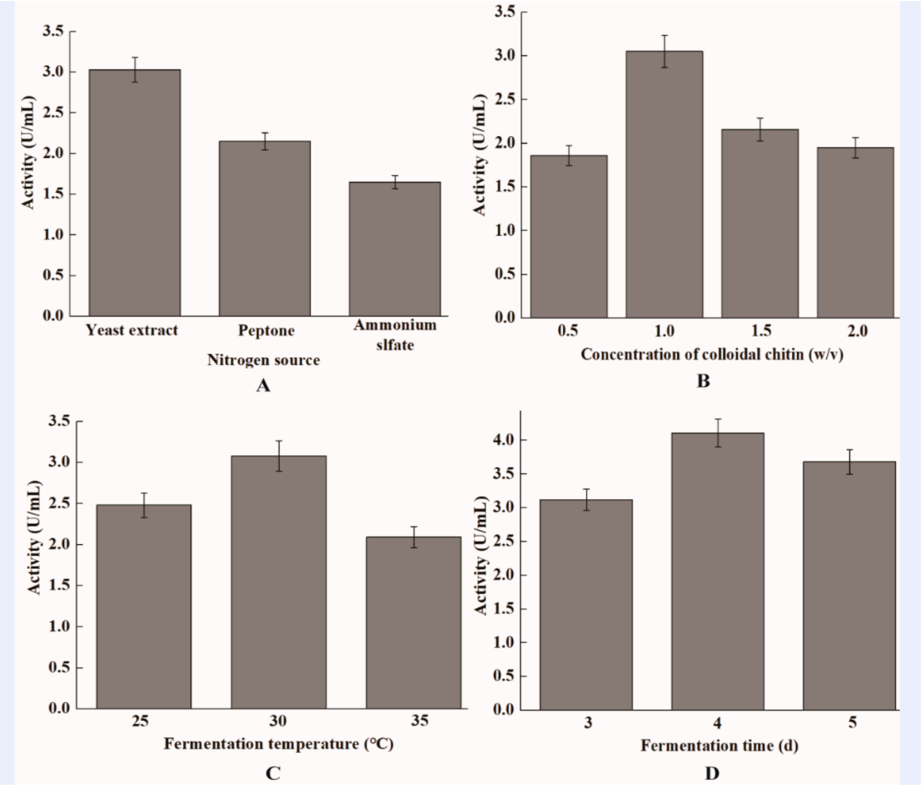

Figure S1. Optimization of nitrogen source (**A**), concentration of colloidal chitin (**B**), fermentation temperature (**C**) and fermentation time (**D**) for production of chitinase from *T. gamsii R*1.

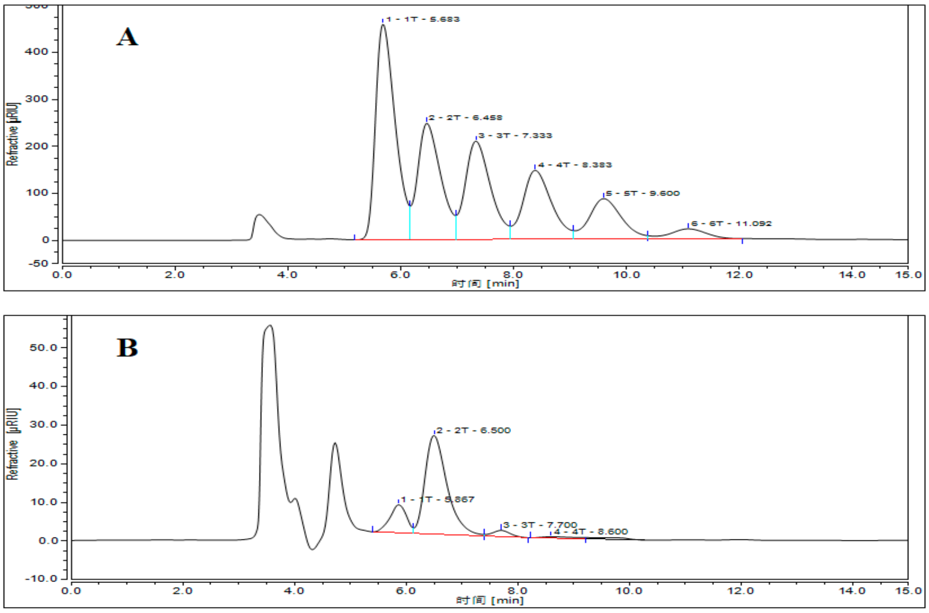

Figure s2. The HPLC charts of standard COSs with degree of polymerization 1 to 6 (**A**) and hydrolysates (**B**). The numbers of 1T to 6T represent GlcNAc, $(GlcNAc)_2$, $(GlcNAc)_3$, $(GlcNAc)_4$, $(GlcNAc)_5$ and $(GlcNAc)_6$, respectively.
